# Validity and reliability of a semi-quantitative food frequency questionnaire for assessing dietary vitamin D and calcium intakes in Iranian childbearing age women

**DOI:** 10.3389/fnut.2022.1028265

**Published:** 2022-10-26

**Authors:** Amir Hossein Moridpour, Maryam Rafraf, Parvin Sarbakhsh, Somayyeh Asghari, Roghayeh Molani-Gol, Mohammad Asghari-Jafarabadi

**Affiliations:** ^1^Student Research Committee, Tabriz University of Medical Sciences, Tabriz, Iran; ^2^Nutrition Research, Department of Community Nutrition, Faculty of Nutrition and Food Science, Tabriz University of Medical Sciences, Tabriz, Iran; ^3^Department of Epidemiology and Biostatistics, School of Public Health, Tabriz University of Medical Sciences, Tabriz, Iran; ^4^Department of Clinical Nutrition, Faculty of Nutritional Sciences and Dietetics, Tehran University of Medical Sciences, Tehran, Iran; ^5^Cabrini Research, Cabrini Health Ltd., Melbourne, VIC, Australia; ^6^Faculty of Medicine, School of Public Health and Preventative Medicine, Nursing and Health Sciences, Monash University, Melbourne, VIC, Australia; ^7^Road Traffic Injury Research Center, Tabriz University of Medical Sciences, Tabriz, Iran

**Keywords:** validity, reliability, food frequency questionnaire, vitamin D, calcium

## Abstract

This study aimed to examine the validity and reliability of a semi-quantitative food frequency questionnaire (FFQ) designed to estimate dietary vitamin D and Calcium (Ca) intakes in a sample of Iranian childbearing age women. An 87-item FFQ was developed and 84 healthy childbearing age women completed it. FFQ was validated by comparing its results with the 24-h dietary recall (24-h recall) and serum 25-hydroxyvitamin D (S-25(OH)D) as the references methods. The FFQ was completed for the second time after 4 weeks to assess the reliability of the questionnaire. Data were analyzed using spearman’s correlation, cross-classification analysis, Bland–Altman plots, the weighted κ, intraclass classification, and the method of triads. Spearman’s correlations between vitamin D and Ca intakes by the FFQ and 24-h recalls and between vitamin D intakes and S-25(OH)D were significant (*r*: 0.706, *r*: 0.959, and *r*: 0.682, respectively, all, *P* < 0.001). Cross-classification for vitamin D and Ca between two dietary methods and for vitamin D intake of FFQ and S-25(OH)D demonstrated that most of the quartiles were categorized into the same or adjacent quartiles. The Bland Altman plots for these nutrients also demonstrated good agreement. For vitamin D, the validity coefficients (VCs) calculated by the method of the triad for three methods were in the range of 0.808–0.843. The weighted κ for agreement of vitamin D and Ca by FFQ1 and FFQ2 were 0.18 and 0.638, respectively. The findings indicated that the developed FFQ has acceptable validity for estimating vitamin D and Ca. Its reliability for Ca was stronger than vitamin D.

## Introduction

Vitamin D, also known as calciferol, is one of the fat-soluble vitamins with two major forms of cholecalciferol (D3) and ergocalciferol (D2) that are found in animals and plant sources, respectively (1). Vitamin D plays a key role in bone health as well as in a number of chronic diseases including heart disease, diabetes, cancer, inflammation, and autoimmune disease (2). Maintenance of optimal vitamin D intake in women of reproductive age can help to ensure normal biological processes relevant to women’s health such as fertility, pregnancy, and lactation (3). It has been shown that low vitamin D level is associated with pre-eclampsia, gestational diabetes, and adverse pregnancy outcomes (4).

Vitamin D deficiency is a common and serious problem in the world, and several studies have shown that about one billion people worldwide have vitamin D deficiency (5). In Iran, it has been reported that more than half of the population suffers from vitamin D deficiency with the highest prevalence among women (6). Iranian women’s clothing due to cultural reasons may be partly involved in their low vitamin D status (7). Thus, their vitamin D intake relies greatly on the food they eat. The recommended daily allowance for vitamin D is 600 IU (15 mcg) per day in women of reproductive ages (8). To ensure that the general population and women of childbearing age consume enough vitamin D, many countries have fortified some foods with vitamin D (9). However, limited foods have been fortified with vitamin D in Iran (10).

Calcium (Ca) is also an essential micronutrient for achieving normal bone development and overall growth (11). Adequate Ca intake promotes mineralization of the skeleton and peak bone mass early in life and maintains bone density during adulthood (12). To achieve maximum Ca balance and maintain good bone health, it is recommended to intake approximately 1,000 mg per day of dietary Ca in 19–50 years old adults (8). Vitamin D promotes Ca absorption in the gut and maintains adequate serum Ca concentrations to enable normal bone mineralization (13). Together with Ca, vitamin D helps protect adults from osteoporosis development and progression as aging (14).

Data on Iranian households’ food intake show that vitamin D and Ca are among the most limiting nutrients in the Iranian diet, mainly due to inadequate dietary intake of these nutrients (15). However, little information is available on vitamin D and Ca intakes among Iranian women. Due to the presence of Ca in many vitamin D-containing foods, it is simple to assess Ca intake with vitamin D intake. Thus, it is necessary to select appropriate methods to assess vitamin D and Ca intake to recognize individuals with an inadequate intake of these nutrients and to evaluate the efficacy of the interventions aiming to improve vitamin D and Ca intake.

Among various dietary evaluation techniques, the food frequency questionnaire (FFQ) has been demonstrated to be a fast, practical, affordable, and effective method for assessing dietary habits over time (16). There is no valid and reliable FFQ to assess dietary intakes of vitamin D and Ca in women of reproductive age in Iran. Therefore, we conducted the present study to develop a semi-quantitative FFQ and assess the validity and reliability of the FFQ to determine vitamin D and Ca intakes in women of reproductive age in Iran.

## Materials and methods

This study was carried out between February and March 2021 on 84 women aged 20–40 years and body mass index (BMI) of <30 kg/m^2^ who were referred to healthcare centers in Kelardasht city (36.4902° N, 51.1426° E) located in northern Iran. At first, the objects and methods of the study were explained to all the participants, and written informed consent was obtained. General information including age, marital status, physical activity/exercise level, and medical history were completed for all eligible individuals. Women who were pregnant or breastfeeding were not included in the study. Following a special diet regimen, smoking, using regular vitamin D and Ca supplements, taking any medication, and having a medical history and/or mental disorders were other exclusion criteria.

To determine the sample size, the correlation coefficient between serum 25-hydroxyvitamin D (S-25(OH)D) level and dietary vitamin D intake was considered based on Itkonen’s study (17) which was estimated to be 84 by considering 95% confidence, 80% power, and two-tailed statistical tests. Multi-stage cluster sampling was performed to recruit the study subjects who were referred to the health centers.

There were three healthcare centers within the Kelardasht city, the study setting. In the first stage of sampling, two out of three health centers were selected at random, and in the second stage, nine sub-centers (rural health houses) were randomly chosen (5 heath houses out of 9 houses from the first health center, and 4 out of 6 health houses from the second center). Finally, in the third stage, 84 eligible participants were randomly selected within the health houses to be a representative sample of the study population. The random sequence was generated by the study statistician utilizing MSExcel2013 software.

The study protocol was approved by the ethical committee of Tabriz University of Medical Sciences (Ethical code: IR.TBZMED.REC.1399.996), Tabriz, Iran.

### Development of food frequency questionnaire

A list of foods that are the main sources of vitamin D and Ca in the Iranian diet was prepared. These food items were selected from the Iranian food composition table and the U.S. Department of Agriculture (USDA). Additionally, vitamin D-fortified products were included in the list. The results of the studies conducted in this field were also checked to ensure that all products containing vitamin D and Ca have been included in our list of foods (17–20). Initially, the developed food list was reviewed by an expert panel including 10 registered dietitians to better assess food sources of vitamin D and Ca. Then, a pilot study was conducted on 20 eligible women other than the study subjects to obtain four 24-h dietary recalls (24-h recalls) from each subject. This pilot study was carried out to identify any new food items containing these two nutrients to be added to the list. Finally, the questionnaire contained 87 food items including 13 items of the cereals, 9 items of legumes, 17 items of the dairy, 6 items of the meat, 5 items of fish and seafood, 12 items of the vegetable, 8 items of the fruits, 6 items of the nuts, 5 items of the oil, and 5 items of the beverages group (Supplementary Table 1). To define the portion size of different foods, a picture booklet of household measures was used. The food list containing a standard portion size for each item was then converted into a semi-quantitative FFQ by asking a question on each food item regarding the frequency of intake (times per day, per week, per month, per year, never or rarely, as appropriate). Then, the reported frequencies were converted to the frequencies per day. Estimates of vitamin D and Ca intake per day were obtained by multiplying frequency per day by the vitamin D and Ca content per gram and weight (gr) of eaten food (based on portion size), and then summing over all intakes. Vitamin D and Ca intake from FFQs (FFQ1 and FFQ2), and 24-h recalls were analyzed using Nutritionist 4 software (First Databank Inc., Hearst Corp., San Bruno, CA, USA).

This first food frequency questionnaire (FFQ1) was completed through a face-to-face interview by a trained dietitian for each individual to assess the relative dietary intake of vitamin D and Ca during the previous 12 months. Since the dietary intake assessment requires high accuracy, direct interviews were preferred to the self-reported method.

### Validity and reliability

For validity assessment, the efficacy of the FFQ1 was compared with the mean dietary intakes obtained from four non-consecutive 24-h recalls as the reference method which comprised 3 weekdays and a weekend with at least 5-day intervals between them. The first 24-h recalls were completed through face-to-face interviews after completing FFQ1 and the remaining were done by phone interviews for 4 weeks. To achieve accurate information about dietary intake, women were asked to report all the foods and beverages that they consumed during the past 24 h, most commonly. The participants were asked not to change their usual dietary patterns during the study duration.

S-25(OH)D levels were also measured as another reference method for validity assessment of the developed FFQ for vitamin D intake using the Enzyme-Linked Immunosorbent assay (ELISA) method (Immundiagnostik AG, Bensheim, Germany, ELISA kit). For this aim, 5 cc venous blood samples were drawn from all participants after 12-h overnight fasting on the day on which the FFQ1 was administered. Blood samples were instantly processed and serum samples were stored at −80°C until the data analysis. S-25(OH)D levels <20 ng/ml were defined as deficient.

In addition, to evaluate the reliability of the developed questionnaire, the FFQ was completed for the second time (FFQ2) 4 weeks apart from FFQ1. The same nutritionist interviewed the same subject in different stages of the study.

### Anthropometry

Weight was measured in light clothing by a calibrated Seca scale to the nearest 0.1 kg. Height was measured without shoes using a Seca stable stadiometer with a precision of 0.5 cm. BMI was reported as the ratio of each person’s weight in kg divided by the height in meters squared.

### Statistical analysis

All statistical analyses were carried out using MedCalc version 20.022. The normal distribution of the data has been ensured by the Kolmogorov–Smirnov test. Data on vitamin D and Ca were non-normally distributed, so medians and ranges were reported.

Spearman’s rank correlation coefficients were used to determine the correlation between vitamin D and Ca intakes by the FFQ, 24-h recall, and S-25(OH)D. To compare the classification of one method vs. another, cross-classification into quartiles of dietary intake of vitamin D and Ca by the FFQ1 and two other methods were performed. The weighted kappa (κ) coefficients with a 95% confidence interval were calculated for the agreement of the quartiles in cross-classification analysis. The categories of κ index mostly follow these definitions: <0, no agreement; 0–0 ⋅ 20, as none to slight; 0.21–0.40, as fair; 0.41–0.60, as moderate; 0.61–0.80, as substantial; and 0.81–1.00, is almost perfect agreement (21). Bland–Altman plots were evaluated to compare agreement between FFQ and the 24-h recall for vitamin D and Ca intakes by mean difference and standard deviation of the difference.

The method of triads is used to examine the correlation between the three measurement methods (22). To calculate the correlation by the triad’s method for vitamin D, three methods [FFQ1, 24-h recalls, and S-25(OH)D] were used and the validity coefficient (VC) for the FFQ1 was calculated using the following equation:


$$\rho \,Q\,I=\sqrt{\frac{r\,Q\,R\times r\,Q\,B}{r\,B\,R}}$$

where the ρQI represents the VC for FFQ1 and true intake, rQR the correlation between FFQ and 24-h recalls, rQB the correlation between FFQ1 and S-25(OH)D, and rBR the correlation between the S-25(OH)D and 24-h recalls. The method is described in detail by Yokota et al. (22). *P* < 0.05 was considered statistically significant.

## Results

In all, 84 women participated in the present study that FFQs, and four 24-h recalls were completed by all of them. The general characteristics of the included participants are summarized in Table 1. The mean age, weight, and BMI were 29.36 ± 5.32 years, 67.45 ± 9.66 kg, and 25.54 ± 3.19 kg/m^2^, respectively. The median of vitamin D and Ca intakes from the FFQ1, FFQ2, and 24-h recalls and S-25(OH)D are presented in Table 2.

**TABLE 1:** Demographic characteristics of the study participants (*n* = 84).

Variables	Mean (SD)
Age	29.36 (5.32)
Weight (kg)	67.45 (9.66)
Height (cm)	162.42 (6.13)
BMI (kg/m^2^)	25.54 (3.19)

***n* (%)**	

Healthy weight (BMI = 18.5–24.9)	35 (41.6)
Overweight (BMI = 25–29.9)	49 (58.4)
**Marital status**	
Married	63 (75)
Single	21 (25)
**Education level**	
High school diploma or below	53 (63.1)
Higher education	31 (36.9)

BMI, body mass index.

**TABLE 2:** Daily vitamin D and Ca intakes assessed by FFQ1, FFQ2, and 24-h recalls and S-25(OH)D (*n* = 84).

	FFQ1	FFQ2	Mean of recalls*	S-25(OH)D (ng/ml)
	Median (IQR)	Median (IQR)	Median (IQR)	Median (IQR)
Vitamin D (μg/day)	4.46 (2.01)	4.64 (1.33)	2.84 (1.61)	20.5 (6.4)
Calcium (mg/day)	930.87 (355.29)	958.22 (364.10)	787.24 (306.00)	–

FFQ1, first administration of FFQ, FFQ2; Second administration of FFQ, S-25(OH)D; Serum level of 25-OH vitamin D, IQR, interquartile range.

*The mean of the four repeated 24-h dietary recalls (The mean of recalls is the average of the four medians).

Vitamin D and Ca intakes in all and 59.5% of subjects were lower than recommended daily dietary allowances, respectively. Levels of S-25(OH)D in 48.8% of women were lower than 20 ng/ml (data not shown).

### Validity study

#### Vitamin D

The results of the correlation coefficients among the three methods are shown in Table 3. Spearman’s correlations between vitamin D intake by the FFQ1, 24-h recalls, and S-25(OH)D were significant (*P* < 0.001). Cross-classification for vitamin D intake between all three methods demonstrated most of the quartiles were categorized into the same or adjacent quartiles. The most misclassification of subjects happened among FFQ1 vs. S-25(OH)D (10.71%).

**TABLE 3:** Cross-classification into quartiles, spearman’s correlations and weighted κ for vitamin D and calcium intake in the validation study (*n* = 84).

	Cross-classification in quartiles			Spearman’s correlations		Weighted	
	Same (%)	same or adjacent (%)	opposite (%)	*r*	*P*	κ	95% CI
FFQ1 vs. 24-h recalls for vitamin D intake	47.61	90.47	9.52	0.706	<0.001	0.504	0.379–0.629
FFQ1 vs. S-25(OH)D	48.8	89.28	10.71	0.682	<0.001	0.504	0.379–0.63
FFQ1 vs. 24-h recalls for Ca intake	80.95	100	0	0.959	<0.001	0.847	0.778–0.916

FFQ1; First administration of FFQ, S-25(OH)D; Serum level of 25-OH vitamin D, 24-h recalls; 24-h dietary recalls.

**FIGURE 1:**
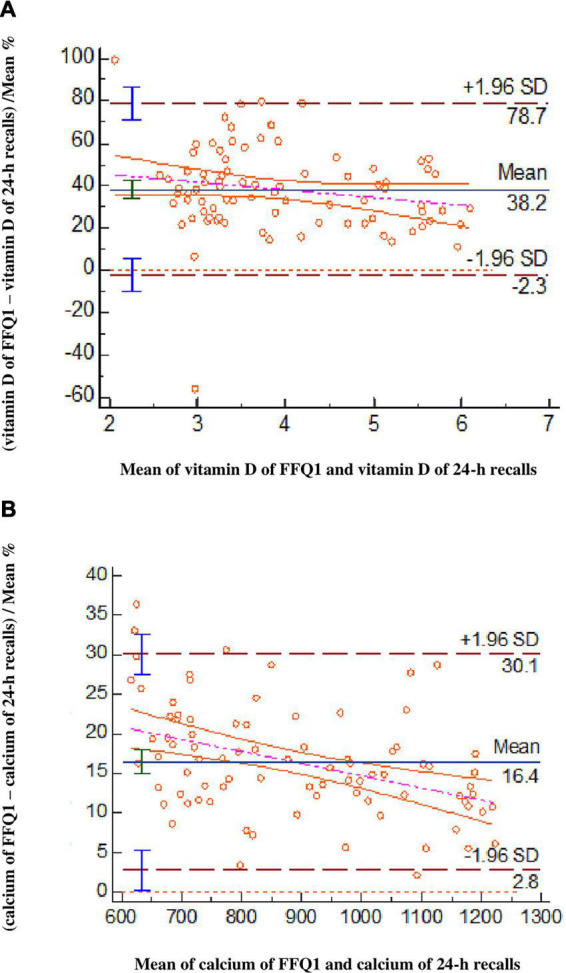
Bland–Altman plots evaluate the agreement between the FFQ1 and 24-h recalls for vitamin D **(A)** and calcium **(B)** intakes (*n* = 84).

The mean difference in vitamin D intake between FFQ1 minus 24-h recalls was 38.2 μg/day and 95% limits of agreement for them were between −2.26 and 78.7, demonstrating that the FFQ1 overestimated the vitamin D intake which is presented in Bland-Altman plots (Figure 1A). The weighted κ was calculated and its value was moderate in FFQ1 vs. S-25(OH)D and FFQ1 vs. 24-h recalls.

**FIGURE 2:**
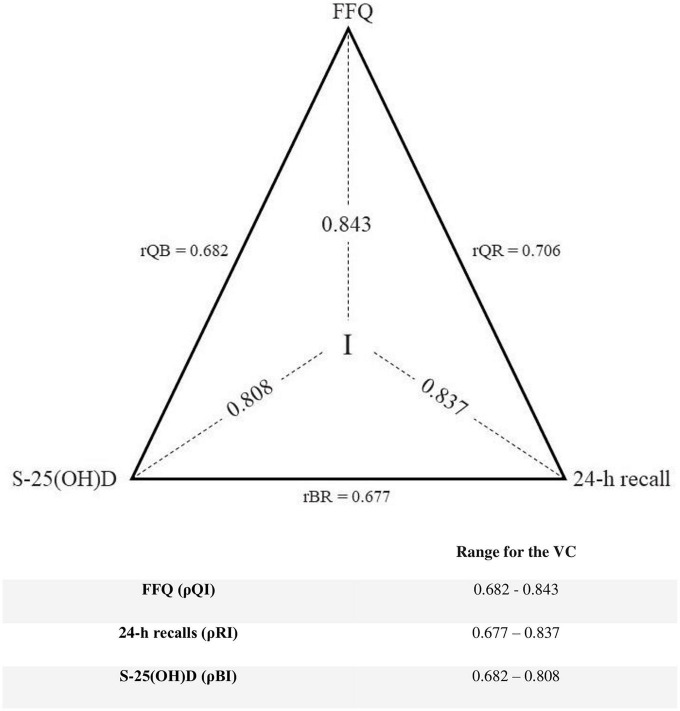
Method of triads: The correlation coefficients (rQR, rRB, rQB) between vitamin D intake estimated by the FFQ1 (Q), the average of four 24-h recalls (R), and the biomarker (B) of vitamin D status [serum 25-hydroxyvitamin D (S-25(OH)D)] and validity coefficient (VC)s (QI, BI, RI) between true intake (I) and estimated intakes. The lower limit is rQB for the FFQ and biomarker, and rBR for the 24-h recalls, while the maximum limit is determined using the triads method (*n* = 84).

To measure agreement (ρQI, ρBI, ρRI) between all three measurements the method of triads was used. The calculated VC by the method of the triad for the FFQ1, 24-h recalls, and S-25(OH)D were 0.843, 0.837, and 0.808, respectively (Figure 2).

#### Calcium

The spearman correlation coefficient was used to calculate the correlation between Ca intake by FFQ1 and 24-h recalls. The results showed a significant relevance among them (*P* < 0.001). The cross-classifications of Ca into quartiles illustrate great agreement and all of the participants are classified into the same or adjacent quartiles. None of the subjects have been into opposite quartiles.

The Bland–Altman analysis revealed that determined Ca intake by FFQ1 had overestimation in comparison to 24-h recalls (Figure 1B). In addition, the value of weighted κ for FFQ1 vs. 24-h recalls represented the high correlation between them.

#### Reliability

Intraclass correlation coefficient first and second administration of FFQ are shown in Table 4. There were significant correlations for vitamin D intake and Ca intake between FFQs (*r*: 0.429 and *r*: 0.875, respectively, *P* < 0.001, for both). The weighted κ for agreement of vitamin D and Ca were 0.18 and 0.638, respectively. The cross-classification quartiles for completed FFQ for the first and second time for vitamin D are as follows: 27.38% of the subjects in the same quartile, 72.61% in the adjacent quartile, and 27.38% in the opposite quartile. Regarding Ca, 57.14% of individuals were classified in the same quartile, 97.62% in the adjacent quartile, and 2.38% in the opposite quartile.

**TABLE 4:** Cross-classification into quartiles and reliability of the FFQ (Intraclass correlation coefficient) (*n* = 84).

	Cross-classification in quartiles			Intraclass correlation coefficient		Weighted	
	Same (%)	same or adjacent (%)	opposite (%)	*r*	*P*	κ	95% CI
FFQ1 v. FFQ2 for vitamin D intake	27.38	72.62	27.38	0.429	<0.001	0.18	0.038–0.323
FFQ1 v. FFQ2 for Ca intake	57.14	97.62	2.38	0.875	<0.001	0.638	0.536–0.739

FFQ1; First administration of FFQ, FFQ2; Second administration of FFQ.

## Discussion

Vitamin D and Ca play vital roles in women’s overall health due to the effect of Ca on bone health and the role of vitamin D in the hemostasis of Ca as well as in women’s reproductive health (23, 24). The prevalence of vitamin D deficiency has been reported to be around 75.1% among Iranian women (25). According to the study findings, 48.8% of participants had vitamin D deficiency. Dietary data evaluated by FFQ1 determined that vitamin D and Ca intakes in all and 59.5% of subjects were lower than recommended daily dietary allowances.

In the current study, an 87-item FFQ was developed to evaluate the dietary intake of vitamin D and Ca in a population of Iranian childbearing-age women. In Iran, numerous studies have assessed the validity of FFQ containing data on several nutrients including vitamin D and Ca. Yet, this is the first study focused on only vitamin D and Ca.

There is no gold standard method for dietary intake assessments, so it is crucial to select a decent reference method to evaluate the validity of the instrument of interest. It should also be noted that the errors of the applied methods need to be as independent of each other as possible (26). In the present study, FFQ1 was validated against 24 h recall method for both vitamin D and Ca and also S-25(OH)D as a biomarker for vitamin D. There are similarities and differences between FFQ and 24-h recalls. Both methods rely on an individual’s perception of portion sizes and memory. Moreover, unlike 24-h recalls, FFQ relies on long-term memory. FFQ uses closed-ended questions, hence, 24-h recalls use open-ended questions (27, 28). Subjects record in detail their food and drink intake during the past 24 h on the 24-h recalls. The food consumed may be coded to a high degree of accuracy using this method. The burden on respondents is minimal and dietary patterns are unaffected by the method (29, 30).

According to the results, a high correlation was seen between estimated vitamin D by FFQ1 and reference methods including 24-h recalls (*r* = 0.706) and S-25(OH)D (*r* = 0.682). There was also a very strong correlation (*r* = 0.959) between amount of Ca intakes from FFQ1 and 24-h recalls. Results of cross-classification for vitamin D between all the three methods [FFQ1, 24-h recalls and S-25(OH)D] and for Ca between two dietary methods (FFQ1 and 24-h recalls) indicated that most of the quartiles were categorized into the same or adjacent quartiles. There was no major misclassification for Ca. The most misclassification of subjects for vitamin D occurred among FFQ1 vs. S-25(OH)D (10.71%). This finding might be a result that S-25(OH)D reflects both dietary intake and vitamin D synthesis induced by UVB radiation in the sunshine. So, S-25(OH)D levels and dietary intake are not always consistent. Thus, persons with low vitamin D intake but having high biogenesis of this vitamin may be classified in the opposite quartile in FFQ1. However, this study was conducted in the winter, when the biogenesis of vitamin D was limited. In addition, women’s sun exposure was also restricted due to traditional dress in the studied community. In such conditions, previous body reserves of vitamin D may contribute most to between-subject variations of S-25(OH)D. Despite this, misreporting in the FFQ1 might be another reason for misclassification in part. Different misclassification rates have been reported in other studies. In a study among Libyan women, the validity of a specific FFQ for vitamin D was validated against 24-h recalls and S-25(OH)D. They reported that the misclassification rates were 2.5 and 7.5% for 24-h recalls and S-25(OH)D, respectively (19). In another study by Masson et al., a 150-item FFQ was validated for some nutrients including vitamin D and Ca intake against a weighted 4-day food record. Misclassification rates using the weighted κ were 0.26 and 0.60 and by the spearman correlation coefficient were 13 and 5% for vitamin D and Ca values, respectively (31). Inconsistent results could be due to using various methods of dietary assessment tools as the reference method, type and number of food items in the designed FFQ, different sample sizes, and stratifying subjects into subgroups.

The current study had a sufficient sample size for Bland-Altman analysis in the comparison of FFQ1 with 24-h recalls. The recommended smallest sample size is 50 (27). Bland-Altman plots demonstrated a satisfactory level of agreement between FFQ1 and 24-h recalls for both vitamin D and Ca. Moreover, Bland-Altman plots demonstrated the overestimation of vitamin D and Ca intakes obtained from FFQ1 in comparison with 24-h recalls, which is a prevalent issue. The FFQ’s overestimation of nutrient intakes could be due to people’s tendency to overrate their intakes when they are required to respond to a large number of food items in an FFQ (32, 33). Subjects might also over report some food items rich in micronutrients, including vitamin D and Ca in order to gain social approval, which consequently biases the FFQ findings (34). It has been also suggested that a 24-h recall might underestimate food intake, which could in turn lead to an overestimation by FFQ (35).

According to the results, the agreement (weighted κ) between vitamin D intakes estimated by FFQ1 vs. 24-h recalls and FFQ1 vs. S-25(OH)D was moderate (weighted κ: 0.504 for both). A possible reason for a moderate agreement between FFQ1 vs. 24-h recalls could be that the main dietary sources of vitamin D are oily fish such as salmon and fortified milk and dairy products (36). Due to the limited number of food items rich in vitamin D, they might not be reported in 24-h recalls, which were applied for 4 weeks. On the other hand, the FFQ represents the dietary intake for the entire previous year. Furthermore, as mentioned before, S-25(OH)D reflects both the intake of vitamin D as well as its sunlight biogenesis. It may explain the moderate agreement between vitamin D intakes estimated by FFQ1 and S-25(OH)D. However, the results of the method of triad indicated that VCs for the three methods are high and nearly similar to each other (0.808 to 0.843). Therefore, it seems that all three methods had a satisfactory agreement in estimating vitamin D intakes. The method of triads assumes that three measurements are linearly correlated, but the random errors between them do not correlate (37–39). The main advantage of this method is the inclusion of the biomarker that presents independent errors in comparison with those of the traditional techniques (22).

For Ca intake data, the value of weighted κ for FFQ1 vs. 24-h recalls represented the high correlation between them (weighted κ: 0.847). It is noteworthy that, in addition to rich dietary sources of Ca including milk and dairy products, several other foods also contain low or moderate amounts of Ca and some of them, like rice and bread were often consumed daily. Thus, the findings of 24-h recalls, which reflect daily intakes during 4 weeks, were strongly correlated with the FFQ for Ca so much.

Altogether, by considering the results of the weighted κ, our designed FFQ has acceptable validity of 0.504 for vitamin D and 0.847 for Ca.

Additionally, measures of agreement between FFQs were estimated for assessing the reliability. There were significant correlations between FFQs in estimating the values of vitamin D and Ca intakes. Of note, most participants for both vitamin D and Ca were classified into the same or adjacent quartiles for both FFQs. Furthermore, Ca had less misclassification between FFQs in comparison with vitamin D. Generally, according to both intraclass correlation coefficient and weighted κ, Ca had more reliability than vitamin D. The higher vitamin D and Ca consumption estimations obtained from the FFQ2 compared to the FFQ1 could also attribute to participants becoming more aware of the study’s purpose and information gathered from 24-h recalls.

Overall, it seems that in the present study, there was wider individual variation in the estimations of vitamin D intake than Ca in the two implemented FFQs. This variation might be due to memory errors in reporting of those food items such as milk and dairy products which had both fortified and non-fortified forms of vitamin D and were included in the list of FFQ. The results of reliability for vitamin D in our study was lower than the study by Zaleha et al. (40) on pregnant women in Malaysia and for Ca was higher than that of the study by Omidvar et al. (20) on Iranian children aged 9–13 years old.

A strength of this validation study was that it was performed in a specific age group of healthy women (childbearing age) with a similar physiological condition including non-pregnant/non-lactation. The subjects were selected from those who attended health centers which represent a suitable sample of community women. To improve the accuracy of reporting intakes, individuals’ dietary intakes from the FFQs were gathered through face-to-face conversation. Also, the 24-h recall approach relies on the participants’ memory and ability to judge portion sizes. This was not considered a flaw in our study because our participants were young, healthy women. In addition to 24-h recalls, S-25(OH)D was used as another reference method for vitamin D. When a biomarker is used as a reference technique, the results are free of recollection bias and misreporting. We did not include subjects using vitamin D supplements to obtain more accurate data on the relationship between dietary intakes of vitamin D and S-25(OH)D.

This study had some limitations as well. The 24-h recall method as a reference method was performed four times during 4 weeks of a season. For precise evaluation of vitamin D intake which has limited food sources, it was more accurate, if 24-h recalls would have been performed during all seasons. The developed FFQ cannot be applicable for other age and BMI ranges of women, and also for pregnant/lactating women. Currently, there is no national or mandatory program for vitamin D and calcium fortification of foods in Iran. A limited number of factories under the supervision of the Ministry of Health enrich some products with vitamin D. The most important of them is milk, which was included in the list of designed FFQ. In case of new fortified foods with vitamin D or Ca enter the market in the future, they should be added to the present designed FFQ and their validity and reliability ought to be evaluated again.

## Conclusion

The developed FFQ had a moderate validity for vitamin D and almost perfect agreement for Ca. Its reliability for Ca was stronger than vitamin D. Vitamin D deficiency and low Ca intake are important health problems in the Iranian population. Therefore, continuous monitoring of their intake status is necessary for screening at-risk subjects and implementing effective proper interventions. This designed FFQ can be used as a low-cost and fast instrument for evaluating vitamin D and Ca intakes in childbearing age women of studied community who can face the health problems associate with the deficiency of these nutrients.

## Data availability statement

The original contributions presented in this study are included in the article/Supplementary material, further inquiries can be directed to the corresponding author.

## Ethics statement

The studies involving human participants were reviewed and approved by the ethical code: IR.TBZMED.REC.1399.996. The patients/participants provided their written informed consent to participate in this study.

## Author contributions

MR designed and conducted the whole project, as a supervisor. AM contributed the data collection and on-site study management. MA-J and PS performed the sampling methodology and statistical analysis under the direction. SA and RM-G provided the advice and consultation. AM and MR wrote the original manuscript and contributed the conception of the manuscript. MR performed the final revision of the manuscript. All authors read and approved the final manuscript.
