# Supplementary material for: Validity and reliability of a semi-quantitative food frequency questionnaire for assessing dietary vitamin D and calcium intakes in Iranian childbearing age women
Source: Front Nutr. 2022 Oct 26;9:1028265. doi: 10.3389/fnut.2022.1028265 (PMC9643800; doi:10.3389/fnut.2022.1028265)
Supplement: Supplementary file 1 [file Table_1.docx]

**Supplementary Table 1.** Food items included in the developed semi-quantitative food frequency questionnaire (FFQ)

| Food item |
| --- |
| 1. Lavash bread 2. Barbari bread 3. Sangak bread 4. French bread 5. Taftoon bread 6. Local bread (containing eggs) 7. Other breads 8. Rice, cooked 9. Barley, cooked 10. Macaroni 11. Biscuits 12. Pastries/Cakes/Cookies 13. Beans, any type 14. Soybean 15. Soybean meal 16. Pea, cooked 17. Split pea, cooked 18. Broad bean, cooked 19. Lentil rice 20. Lentil soup (Adasi) 21. Low fat milk fortified with vitamin D 22. Full-fat milk fortified with vitamin D 23. Low fat pasteurized milk 24. Full-fat pasteurized milk 25. Soy milk 26. Chocolate milk 27. Low fat pasteurized yogurt 28. full fat pasteurized yogurt 29. Low fat pasteurized cheese 30. full-fat pasteurized cheese 31. Pizza cheese 32. Cream cheese 33. Kashk (liquid/dried) 34. Gharaghoroot 35. Cream 36. Ice cream 37. Doogh (yogurt drink) 38. Egg 39. Red meat 40. Ground meat 41. Chicken 42. Turkey meat 43. Beef or sheep liver 44. Trout 45. Salmon fish 46. Any other type of fish (fresh or frozen) 47. Canned fish 48. Shrimp 49. Mushrooms, cooked 50. Tomato 51. Cucumber 52. Carrot 53. Lettuce 54. Cabbage (white/red/broccoli/brussel sprout) 55. Cauliflower 56. Spinach (raw/cooked) 57. Green leafy vegetables, raw 58. Green leafy vegetables, cooked 59. Green Pea 60. Potato 61. Onion 62. Orange 63. Orange juice 64. Bitter orange 65. Apple 66. Tangerine 67. Dates 68. Kiwi 69. Dried fruits 70. Chocolate/bars 71. Walnuts 72. Almonds 73. Peanuts 74. Pistachios & hazelnuts 75. Seeds (sunflower, watermelon, pumpkin) 76. Sesame 77. Margarine 78. Butter 79. Vegetable oil/Sunflower oil 80. Mutton tallow/Beef tallow 81. Mayonnaise sauce 82. Ketchup 83. Mineral water 84. Drinking water 85. Carbonated soft drinks 86. Non-alcoholic malt drinks 87. Tea |
